# Transcriptional characterization and response to defense elicitors of mevalonate pathway genes in cotton (*Gossypium arboreum* L.)

**DOI:** 10.7717/peerj.8123

**Published:** 2019-11-20

**Authors:** Zhiqiang Zhang, Wei Liu, Zongbin Ma, Wei Zhu, Lin Jia

**Affiliations:** Collaborative Innovation Center of Henan Grain Crops/Agronomy College, Henan Agricultural University, Zhengzhou, China

**Keywords:** *Gossypium arboreum*, Terpene biosynthesis, MVA pathway, Expression profile, Elicitor response

## Abstract

The mevalonate (MVA) pathway is responsible for the biosynthesis of cytosolic terpenes including gossypol and its derivatives, which play an important role in the cotton plant’s defense against pathogens and herbivores. In this study, we identified and cloned 17 potentially functional genes encoding enzymes that catalyze the six steps of the MVA pathway in *Gossypium arboreum*. Expression pattern analysis by qRT-PCR demonstrated that these genes had tissue-specific expression profiles and were most prevalently expressed in roots. Moreover, these genes were up-regulated in response to several elicitors, including methyl jasmonate and salicylic acid, as well as *Verticillium dahliae* infection and *Helicoverpa armigera* infestation. This indicates that the MVA pathway genes are involved in the signaling pathway regulated by exogenous hormones and the resistance of cotton plants to pathogens and herbivores. Our results improve the understanding of cytosolic terpene biosynthesis in *Gossypium* species and lay the foundation for further research on gossypol biosynthesis.

## Introduction

Terpenes, also known as isoprenoids, are the largest class of natural compounds composed of two isomeric 5 carbon skeletons known as isopentenyl diphosphate (IPP) and dimethylallyl diphosphate (DMAPP), and they are ubiquitous in nature with a diverse range of structures and functions (Lange et al., 2000). Terpenes are classified based on the number of C5 units in their structure as follows: hemiterpenes (C5), monoterpenes (C10), sesquiterpenes (C15), diterpenes (C20), sesterterpenes (C25), triterpenes (C30), tetraterpenes (C40), and polyterpenes (>C40) (Ashour, Wink & Gershenzon, 2010). Terpenes are vital to the growth and development of plants by participating in their primary metabolism, such as phytohormones (abscisic acid, cytokinins, gibberellins, and brassinosteroids), photosynthetic pigments (chlorophylls and carotenoids), electron carriers (plastoquinones and ubiquinones), and membrane components (steroids) (Liu et al., 2005; Tetali, 2019). A majority of plant terpenes are involved in secondary metabolism and serve primarily in ecological roles as a response to biotic and abiotic factors. For example, some terpenes act as phytoalexin defenses against pathogens or herbivores and can induce adjacent plants to begin defense responses (Ahuja, Kissen & Bones, 2012; Aljbory & Chen, 2018; Arimura et al., 2000; Wittstock & Gershenzon, 2002). When plants are flowering, some low molecular mass terpenes are released to attract pollinating insects for pollination (Pansarin, Bergamo & Ferreira-Caliman, 2018; Pichersky & Gershenzon, 2002). Terpenes can also work as allelopathic agents to inhibit or promote seed germination and seedling growth (Kato-Noguchi et al., 2017). As well as the important role of terpenes in plants, many also have high commercial value and are widely used in the pharmaceutical, flavor and fragrance, and biofuel industries (Jessica Elizabeth et al., 2017; Mewalal et al., 2017; Weaver, 2014; Zyad et al., 2018).

**Figure 1:**
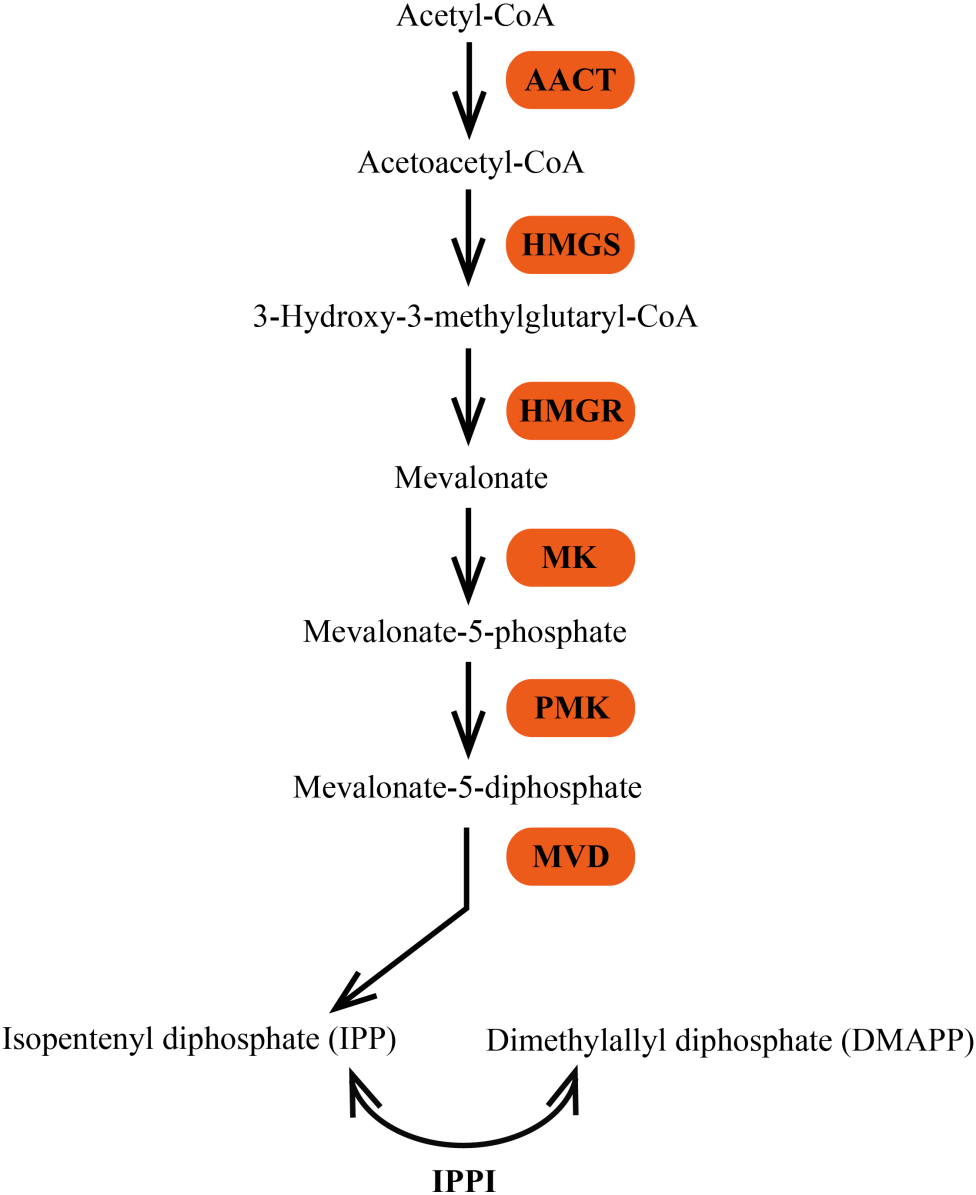
The MVA pathway of biosynthesis of terpene precursors IPP and DMAPP.

In plants, terpenes are derived from the common precursor IPP and its isomer DMAPP, which are synthesized by two independent pathways: the mevalonate (MVA) pathway in the cytosol and the 2-*C*-methyl-D-erythritol 4-phosphate (MEP) pathway in the plastid (Bick & Lange, 2003; Laule et al., 2003). The MVA pathway is generally considered to synthesize precursors for the formation of sesquiterpenes, triterpenes, and sterols in the cytosol or transport to mitochondria for ubiquinone biosynthesis (Aharoni, Jongsma & Bouwmeester, 2005). Six enzymes are involved in the MVA pathway (Fig. 1). The initial reaction of the MVA pathway is that two molecules of acetyl-CoA are catalyzed by acetoacetyl-CoA thiolase (AACT; EC 2.3.1.9) to yield acetoacetyl-CoA, which is then converted to 3-hydroxy-3-methylglutaryl-CoA (HMG-CoA) by HMG synthase (HMGS; EC 2.3.3.10). The third step of the MVA pathway is the conversion of HMG-CoA to MVA, which is catalyzed by the enzyme 3-hydroxy-3-methylglutaryl-CoA reductase (HMGR; EC 1.1.1.34). MVA is then phosphorylated to mevalonate-5-diphosphate in two successive reactions catalyzed by mevalonate kinase (MK; EC 2.7.1.36) and phosphomevalonate kinase (PMK; EC 2.7.4.2). The last step of IPP biosynthesis is an ATP-dependent decarboxylation of mevalonate-5-diphosphate, which is catalyzed by mevalonate diphosphate decarboxylase (MVD; EC 4.1.1.33) (Newman & Chappell, 1999).

MVA pathway genes have been identified and characterized in several plants, such as *Hevea brasiliensis* (Sando et al., 2008), *Picrorhiza kurroa* (Pandit et al., 2013), and *Tripterygium wilfordii* (Liu et al., 2016). Additionally, many studies have focused on a specific gene in the MVA pathway. AACT is encoded by a small gene family in plants (Carrie et al., 2007; Pereto, Lopez-Garcia & Moreira, 2005). In *Arabidopsis thaliana*, the T-DNA insertion *aact2* mutant has an embryo-lethal phenotype, indicating that *AtAACT2* has an essential role in terpene biosynthesis (Jin, Song & Nikolau, 2012). HMGS is an important condensing enzyme and is one of the most extensively studied in the MVA pathway. For example, the overexpression of *Brassica juncea* HMGS in *A. thaliana* enhances the sterol content and stress tolerance in transgenic plants (Wang et al., 2012). HMGR, which is a rate-limiting enzyme in the classical MVA pathway, is by far the most studied enzyme (Rodríguez-Concepción, 2006). It was shown to have key regulatory functions in the biosynthesis of various terpene end products and to respond to a variety of external stimuli including light, herbicide treatment, wounding, pest attacks, and heavy metal exposure as well as endogenous elements such as protein factors, phytohormones, *trans*-farnesol, phytosterols, sphingolipids, protein kinases, and Ca^2+^ (Hemmerlin, Harwood & Bach, 2012). MK, PMK, and MVD catalyze the final three steps of the MVA pathway, however relatively few studies have been conducted on them. *Catharanthus roseus MK*, *PMK*, and *MVD* genes functionally complement the corresponding yeast MVA pathway deletion mutants (Simkin et al., 2011). There are also many studies showing that the MVA pathway is involved in plant defense and symbiotic signaling. Transgenic *Arabidopsis* plants overexpressing mutant *BjHMGS1* (S359A) display an enhanced tolerance to *Botrytis cinerea* over the vector-transformed *Arabidopsis* (Wang et al., 2012)*. MtHMGR1* is directly involved in the signaling pathway that transduces endosymbiotic microbial signals in *Medicago truncatula* (Venkateshwaran et al., 2015).

Cotton fiber is the most important renewable textile fiber, making it an economically valuable crop. Cottonseed is an important source of edible oil, industrial oil, and feed as it is rich in oil and proteins. However, gossypol toxicity limits the utilization of cottonseed productions. Gossypol is a sesquiterpene uniquely synthesized in the cytosol through the MVA pathway by *Gossypium* species; it functions as a phytoalexin in the defense against pathogens and herbivores (Tian et al., 2016). Furthermore, gossypol also has important application value in the field of medical and health care, it can be used as a male contraceptive and is a potential cell proliferation inhibitor in various types of cancers (Coutinho, 2002; Hsiao et al., 2012; Zeng et al., 2019). The research on the genes involved in gossypol biosynthesis is the basis of developing the cotton with gossypol-free seed and normal gossypol content in other tissues by genetic engineering (Ma et al., 2016). Two *HMGR* genes (*hmg1* and *hmg2*) have been identified in *G. hirsutum*, and expression pattern analysis indicated that *hmg1* is constitutively expressed, while *hmg2* is highly expressed in roots and fibers and may be involved in the sesquiterpenoid biosynthesis in developing embryos (Loguercio et al., 1999). In both *G. hirsutum* and *G. barbadense*, the *HMGR* gene is able to be induced by *Verticillium dahlia*, whereas resistant *G. barbadense* react more rapidly (Joost et al., 1995). A unique conserved gene cluster containing four *HMGR* genes has been found in *Gossypium* species (Liu et al., 2018). However, though several investigations have been carried out related to isolation, cloning and characterization of the MVA pathway genes in cotton, the response to elicitors including chemicals and biofactors is still unknown. It has been reported that methyl jasmonate-treated can increase the production of gossypol (Frankfater, Dowd & Triplett, 2009), therefore studies on these elicitors can contribute to regulate gossypol synthesis.

In this study, we identified the MVA pathway genes in *G. arboreum* at the genome-wide level. Then, we detected the expression levels of these genes in diverse cotton tissues. Additionally, cotton seedlings were treated with methyl jasmonate (MeJA), salicylic acid (SA), *Verticillium dahliae* infection, and *Helicoverpa armigera* infestation, and the post-treatment expression levels of the MVA pathway genes were determined. Our results provide the basis for further investigations into the roles of the MVA pathway genes in cotton terpene biosynthesis.

## Materials and Methods

### Sequence retrieval and annotation of the MVA pathway genes

The *G. arboreum* genome data (Du et al., 2018) was downloaded from the CottonGen database (https://www.cottongen.org/). The protein sequences of the *Arabidopsis* MVA pathway genes were acquired from the TAIR10 database (http://www.arabidopsis.org) (Tholl & Lee, 2011), and used as queries to search the *G. arboreum* genome data with the BlastP and tBlastN programs. All hits were subjected to the Pfam database (El-Gebali et al., 2019) to confirm the presence of conserved domains. The InterPro database (Mitchell et al., 2019) was applied to further determine each candidate member of the MVA pathway genes. The theoretical molecular weight (Mw) and isoelectric point (pI) of each protein were inferred using the ProtParam tool (https://web.expasy.org/protparam/).

### Chromosomal localization and analysis of genetic variations

The physical chromosome locations of the MVA pathway genes were retrieved from the *G. arboreum* genome annotation data and visualized using MapInspect software (Ralph van Berloo, Wageningen, Netherlands) (Liu et al., 2015) according to their gene starting positions and chromosomal lengths. The whole genome re-sequencing data for 215 *G. arboreum* accessions were downloaded from https://www.ncbi.nlm.nih.gov/bioproject/PRJNA349094. Single nucleotide polymorphisms (SNPs) were detected in each MVA pathway gene and within sequences 5 kb upstream and downstream, and exonic and intronic SNPs of each gene were counted. The SNP density was calculated by dividing the number of SNPs in a designated region by the length.

### Plant materials and treatments

*Gossypium arboreum* acc. Shixiya 1 was used in this study to clone the MVA pathway genes and conduct gene expression analyses; the seeds were supplied by the Institute of Cotton Research, Chinese Academy of Agricultural Sciences (CAAS, Anyang, China). For tissue-specific expression profiling, roots, stems, cotyledons, and leaves were harvested from 2-week-old seedlings grown in a greenhouse. Developing ovules were collected at 0, 10, 20, 30, and 40 days post anthesis (DPA). Cotton seeds were sown in sand, incubated for about 12 days, and the seedlings were transferred to a liquid culture medium in a growth chamber at 28 °C with a 16-h light/8-h dark photoperiod until the third true leaf appeared (Li et al., 2018a). For phytohormone treatments, seedlings were irrigated with 100 µM MeJA or 2 mM SA (Li et al., 2018b; Shah et al., 2013), after which the roots were harvested at 0, 1, 3, 6, and 12 h. Seedlings treated with the same volume of absolute ethanol were used as mock controls. For *V. dahliae* infection, seedlings were inoculated with 1 × 10^7^ spores of *V. dahliae* strain V991 using the root-dip method (Zhang et al., 2012), and the roots were harvested at 0, 6, 12, 24, and 48 h after treatment. For insect infestation, a third instar larva of *H. armigera* (Hübner) was released on each true leaf on the cotton plants after 6 h of starvation (Huang et al., 2015), and the rest of leaves were sampled from the infested plants at 0, 6, 12, 18, and 24 h. Seedlings grown in normal conditions were used as mock controls for the *V. dahliae* infection and insect infestation. The mock samples were collected at the same time point as each treatment. Three biological repeats were performed for each experiment. All samples were quick-frozen in liquid nitrogen and stored at –80 °C until RNA extraction.

### RNA isolation and cDNA synthesis

Total RNA was extracted from each sample using the RNA Extraction Kit (TIANGEN, Beijing, China). The NanoDrop2000 microvolume spectrophotometer (NanoDrop Technologies, Wilmington, DE, USA) was employed to determine the RNA concentration, and the RNA integrity was analyzed by 1.5% agarose gel electrophoresis (Wang et al., 2017). The first-strand cDNA was synthesized from 1 µg total RNA using the PrimeScript™ 1st Strand cDNA Synthesis Kit (TaKaRa, Dalian, China).

### Cloning of full-length cDNAs in the MVA pathway

Based on predicted sequences of the MVA pathway genes in *G. arboreum*, we designed gene-specific primers using Oligo software (Version 7.60, Molecular Biology Insights, Cascade, CO, USA) (Rychlik, 2007) to amplify genes with complete open reading frames (ORFs) (Table S1). The template cDNA was derived from a mixed sample of nine tissues, including roots, stems, cotyledons, leaves, and ovules at 0, 10, 20, 30, and 40 DPA. Reverse transcription PCR (RT-PCR) reactions were performed using Tks Gflex™ DNA Polymerase (TaKaRa, Dalian, China) with the following program: 94 °C for 1 min, then 35 cycles of 98 °C for 10 s, 60 °C for 15 s, and 68 °C for 2 min. PCR products were purified with the MiniBEST Agarose Gel DNA Extraction Kit (TaKaRa, Dalian, China), cloned into the pMD18-T cloning vector (TaKaRa, Dalian, China), and transformed into *Escherichia coli* DH5 *α* for massive sequencing.

### Quantitative real-time PCR

Quantitative real-time PCR (qRT-PCR) was performed to analyze the expression of the MVA pathway genes in *G. arboreum.* Amplification reactions were performed on the LightCycler 480 system (Roche, Basel, Switzerland) using SYBR^^®^^ Premix Ex Taq™ (TaKaRa) with the following parameters: 95 °C for 30 s, followed by 40 cycles of 95 °C for 5 s and 60 °C for 30 s. A melting curve was generated from 60 °C to 95 °C to assess the specificity of target sequences. Specific primers are listed in Table S2 and cotton *UBQ7* was used as an internal control (Cui et al., 2017). The 2^−^^Δ^^*C*^^t^ method was used to calculate the relative expression levels of the MVA pathway genes (Schmittgen & Livak, 2008). ΔCt was calculated by subtracting the Ct values of *UBQ7* (internal control) with the target gene within the same sample. For tissue expression profiling, 2^−^^Δ^^*C*^^t^ values were used for one-way ANOVA with Tukey’s HSD test using SPSS software (Version 21.0, IBM Corporation, Chicago, IL, USA) to assess the significant differences among the various tissues. For the four treatments, 2^−^^Δ^^*C*^^t^ values were used for Student’s *t*-test to assess the significant differences between the treated and untreated (mock) samples. Finally, the results were visualized using the Origin software (Version 8.0, OriginLab, Northampton, Massachusetts, USA) (Li et al., 2018a).

## Results

### Identification of the MVA pathway genes in *G. arboreum*

To identify the MVA pathway genes in *G. arboreum*, the BlastP and tBlastN programs were utilized to search against the recent *G. arboreum* genome data (Du et al., 2018) with the query sequences from *Arabidopsis*. All candidate genes were submitted to the Pfam and InterPro databases to confirm members of each gene family of the MVA pathway. Next, the full-length cDNA of each MVA pathway gene was cloned to determine the sequence. As a result, we identified two *AACT* genes, three *HMGS* genes, nine *HMGR* genes, one *MK* gene, one *PMK* gene, and one *MVD* gene in *G. arboreum*. Additionally, we found an *MK* gene locus with a short predicted protein sequence and no evidence of expression was found by RT-PCR in various tissues of *G. arboreum*, suggesting that it may have become a pseudogene. This gene was therefore not included in the following analyses. The *HMGS*, *HMGR*, *MK*, *PMK*, and *MVD* genes have been identified in *G. raimondii* (Liu et al., 2018), and based on the orthologous relationship between *G. arboreum* and *G. raimondii*, the genes were named *GaHMGS1*-*3*, *GaHMGR1*-*9*, *GaMK1* and *GaMK2*, *GaPMK*, and *GaMVD* with the same numbering as those in *G. raimondii*. The *AACT* genes were named *GaAACT1* and *GaAACT2* based on the order of the corresponding chromosome locations (Tables S3 and S4).

**Figure 2:**
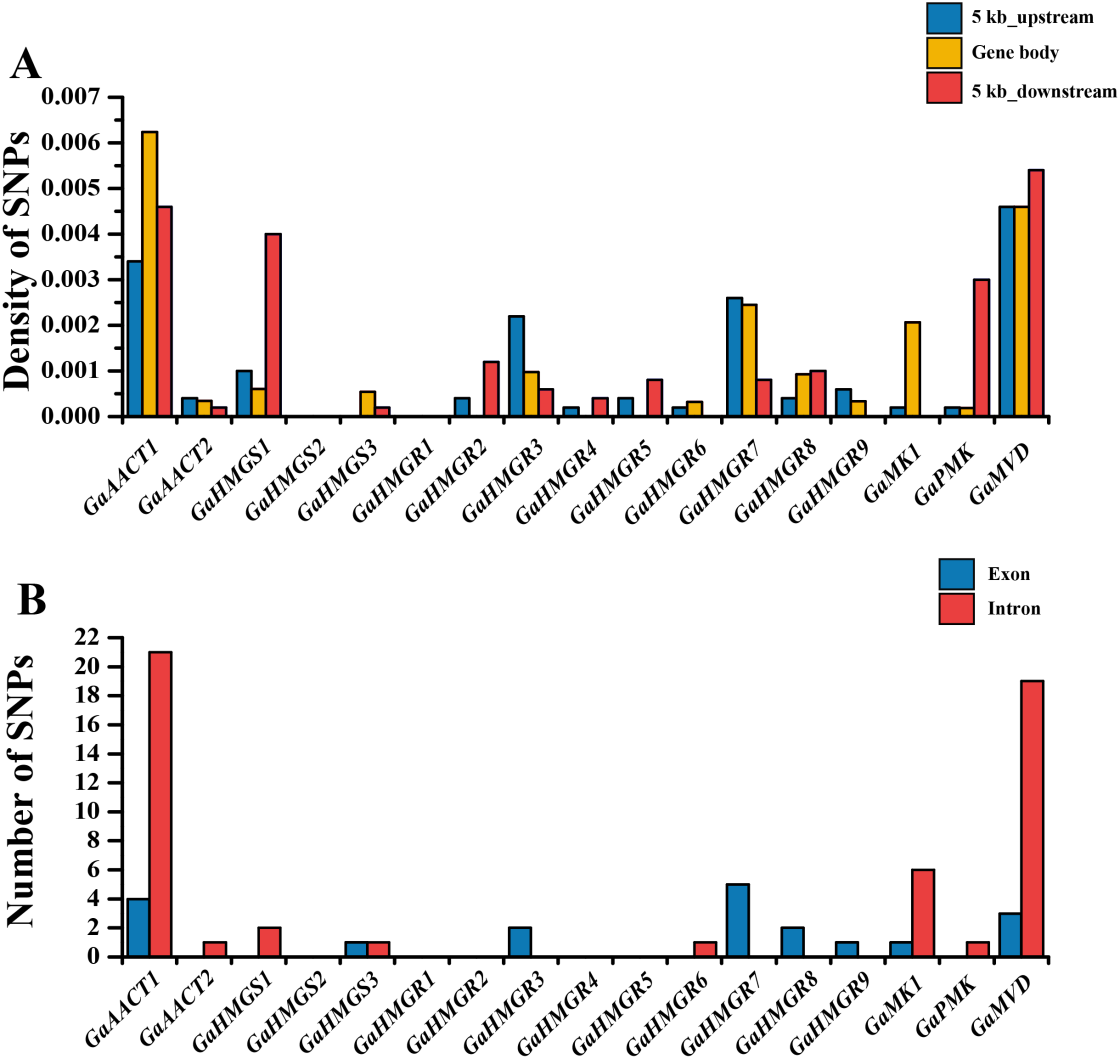
Genetic variations in *G. arboreum* MVA pathway genes. (A) SNP density of the gene body and sequences 5 kb upstream and downstream of each MVA pathway gene in 215 *G. arboreum* accessions. Gene body is defined as the entire genomic sequence of each gene from the transcription start site to the end of the transcript. (B) Number of SNPs in exons and introns of each MVA pathway gene.

The chromosomal distributions of *G. arboreum* MVA pathway genes were analyzed and the 18 genes were shown to be distributed on eight chromosomes (Fig. S1). Three genes each were detected on chromosomes 1, 11, and 12, and two genes were present on chromosome 13, whereas only a single gene was localized on chromosomes 4, 6, and 8. Additionally, there was an *HMGR* gene cluster containing four genes (*GaHMGR2*, *GaHMGR3*, *GaHMGR4*, and *GaHMGR5*) on chromosome 3. To investigate the conservation of the MVA pathway genes, we calculated the SNP density in the gene body as well as in sequences 5 kb upstream and 5 kb downstream of each MVA pathway gene using genome re-sequencing data (Fig. 2A and Table S5). Five genes (*GaHMGS2*, *GaHMGR1*, *GaHMGR2*, *GaHMGR4*, and *GaHMGR5*) lacked SNPs in the 215 *G. arboreum* lines. The SNP density of *GaAACT1*, *GaHMGS3*, *GaHMGR6*, and *GaMK1* gene body was higher than both the 5 kb upstream and 5 kb downstream sequences, while *GaHMGS1* was lower than both of them. *GaAACT2*, *GaHMGR3*, *GaHMGR7*, and *GaHMGR9* had an SNP density lower than the 5 kb upstream but higher than the 5 kb downstream sequences, and *GaHMGR8* was the opposite. The SNP density of *GaPMK* and *GaMVD* was the same as the 5 kb upstream, but lower than the 5 kb downstream. The number of SNPs in the exons of most MVA pathway genes was lower than that in the introns (Fig. 2B and Table S6).

### Expression profiles of the MVA pathway genes in various *G. arboreum* tissues

**Figure 3:**
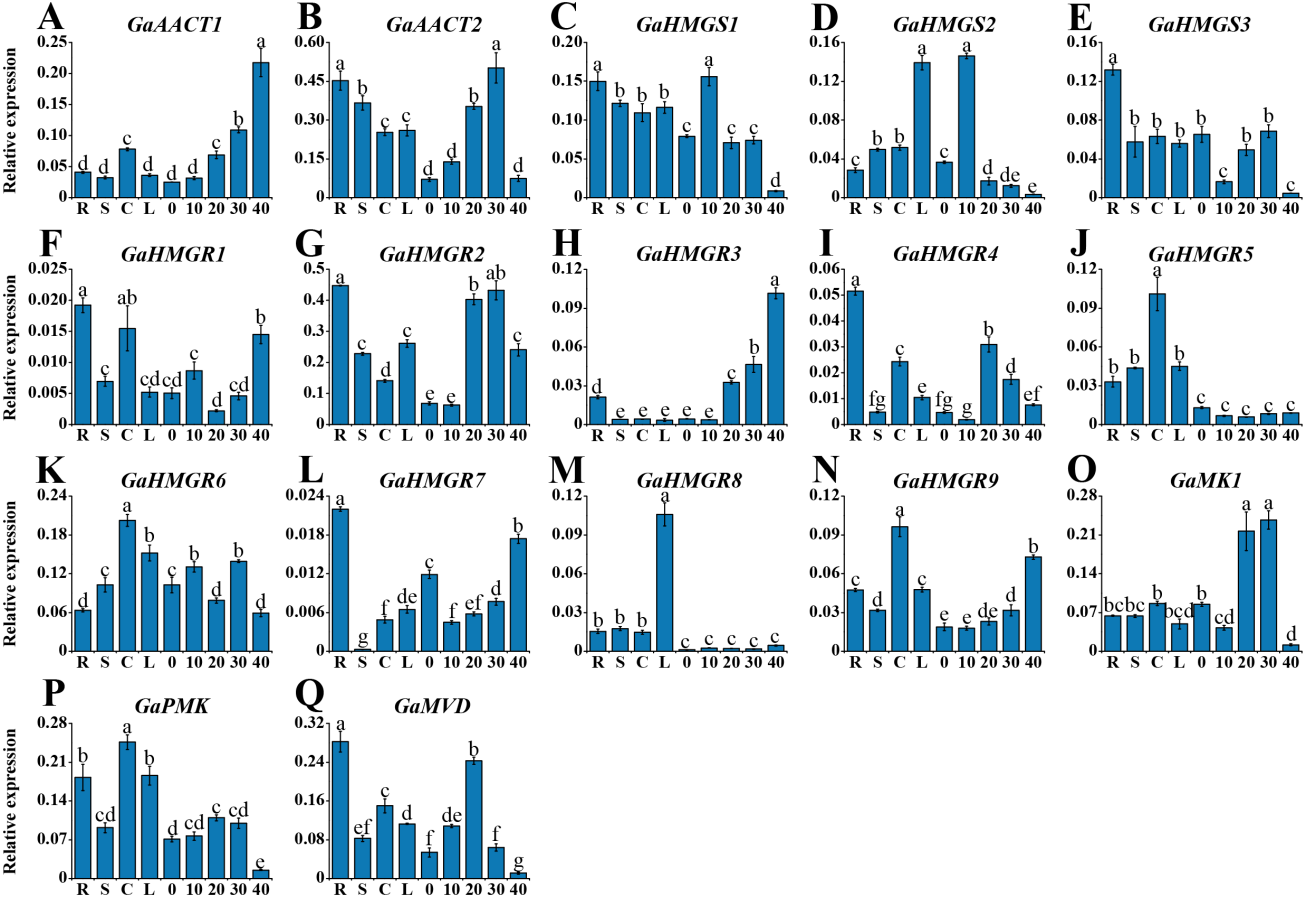
Expression profiles of the MVA pathway genes in various *G. arboreum* tissues. (A) *GaAACT1*. (B) *GaAACT2*. (C) *GaHMGS1*. (D) *GaHMGS2*. (E) *GaHMGS3*. (F) *GaHMGR1*. (G) *GaHMGR2*. (H) *GaHMGR3*. (I) *GaHMGR4*. (J) *GaHMGR5*. (K) *GaHMGR6*. (L) *GaHMGR7*. (M) *GaHMGR8*. (N)*GaHMGR9*. (O) *GaMK1*. (P) *GaPMK*. (Q) *GaMVD*. Relative expression levels in roots (R), stems (S), cotyledons (C), leaves (L), and ovules at 0, 10, 20, 30, and 40 DPA were calculated by the 2^−ΔCt^ method with cotton *UBQ7* as an internal control. Error bars represent the standard deviations estimated from three independent biological replicates. Statistical analysis was performed using one-way ANOVA with Tukey’s HSD test. The maximum value is marked as ‘a’, then the same letter indicates that the difference is not significant (*p* > 0.05), and the different letters indicate significant differences (*p* < 0.05).

To investigate the tissue-specific expression profiles of the MVA pathway genes, we detected their expression in various tissues of *G. arboreum* acc. Shixiya 1, including roots, stems, cotyledons, leaves, and developmental ovules at 0, 10, 20, 30, and 40 DPA (Table S7). As indicated in Fig. 3, the MVA pathway genes exhibited diverse expression profiles in different tissues. *GaAACT1* expression in the ovules increased with development and showed the highest expression level at 40 DPA. *GaAACT2* was highly expressed in the roots, stems, and ovules at 20 and 30 DPA. The expression levels of *GaHMGS1* and *GaHMGS3* were relatively high in the roots, but *GaHMGS1* expression was high in the ovules at 10 DPA, while *GaHMGS3* was low. However, *GaHMGS2* expression was high in the leaves and ovules at 10 DPA. Several *GaHMGR* genes had relatively high expression levels in the roots except *GaHMGR6*, which was highly expressed in the cotyledons, leaves and ovules at 10 and 30 DPA. *GaHMGR1*, *GaHMGR2*, *GaHMGR4*, and *GaHMGR7* had the highest expression in the roots, while *GaHMGR3*, *GaHMGR5*, *GaHMGR8*, and *GaHMGR9* expressed moderately in the roots. The expression level of *GaHMGR1* was also relatively high in the cotyledons and ovules at 40 DPA. *GaHMGR2*, *GaHMGR3*, and *GaHMGR4* were expressed at moderate levels in the late stages of ovule development, whereas *GaHMGR5*, *GaHMGR8*, and *GaHMGR9* were mainly expressed in the seedling stage. *GaMK1* was predominantly expressed in the ovules at 20 and 30 DPA. However, *GaPMK* was highly expressed in seedling tissues. *GaMVD* showed a preferential expression in the roots and ovules at 20 DPA.

### Expression analysis of the MVA pathway genes in response to MeJA

**Figure 4:**
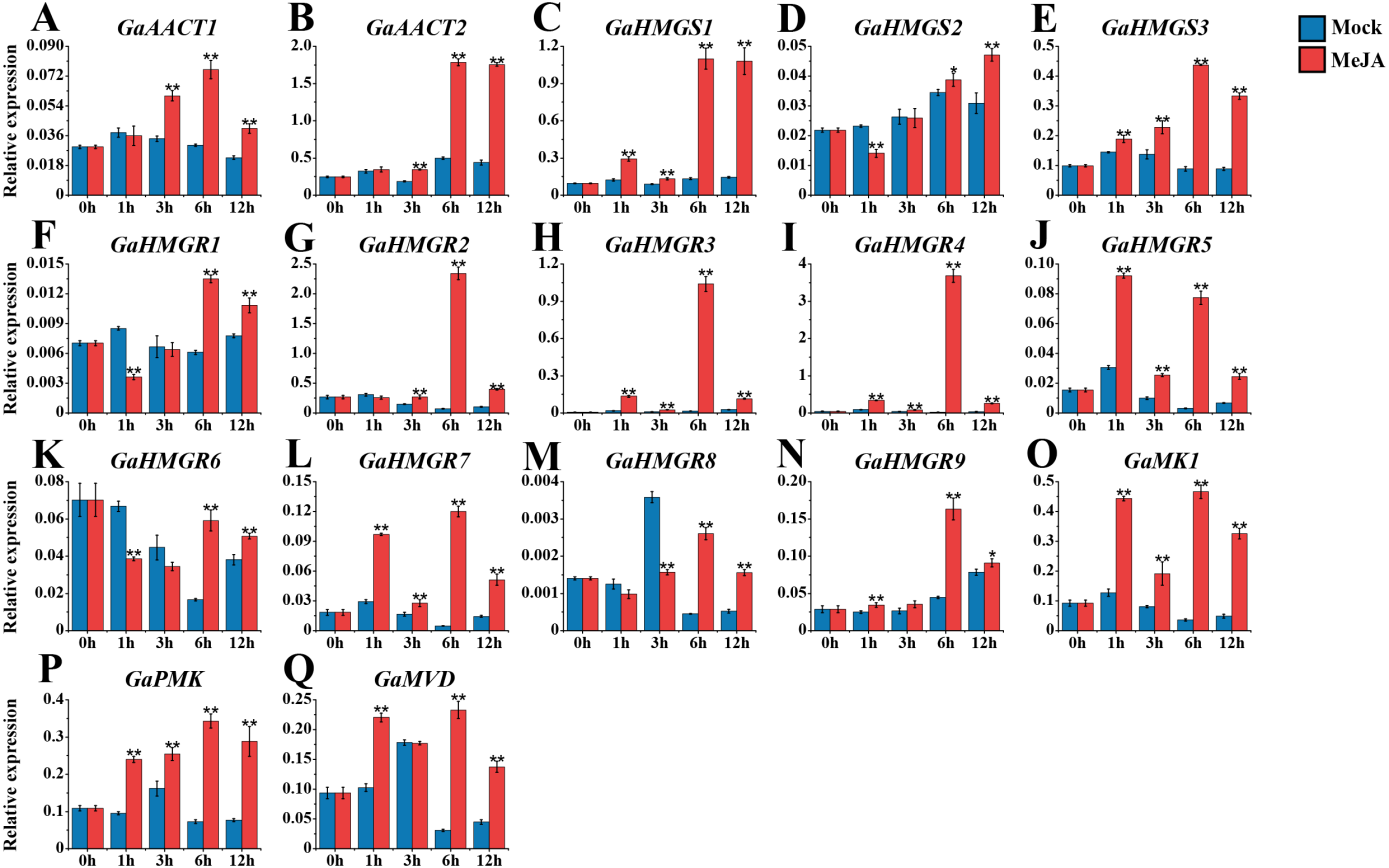
Expression analysis of the MVA pathway genes in response to MeJA. (A) *GaAACT1*. (B) *GaAACT2*. (C) *GaHMGS1*. (D) *GaHMGS2*.** (E) *GaHMGS3*. (F) *GaHMGR1*. (G)* GaHMGR2*. (H) *GaHMGR3*. (I) *GaHMGR4*. (J) *GaHMGR5*. (K) *GaHMGR6*. (L)* GaHMGR7*.** (M) *GaHMGR8*. (N)* GaHMGR9*. (O) *GaMK1*.** (P) *GaPMK*. (Q) *GaMVD*. Relative expression levels were calculated by the 2^−ΔCt^ method with cotton *UBQ7* as an internal control. Error bars represent the standard deviations estimated from three independent biological replicates. Asterisks indicate significant differences between the treated and mock samples, ^∗^*P* ≤ 0.05, ^∗∗^*P* ≤ 0.01, Student’s *t*-test.

The expression levels of the MVA pathway genes were detected after 100 μM MeJA treatment (Fig. 4 and Table S8). The expression levels of most MVA pathway genes were up-regulated and reached a peak at 6 h after induction. *GaAACT1* and *GaAACT2* expression was significantly up-regulated between 3–12 h compared with the mock control. The expression levels of *GaHMGS1* and *GaHMGS3* began to increase significantly after 1 h of MeJA treatment and peaked at 6 h. However, *GaHMGS2* expression was down-regulated at 1 h after treatment, increased at 6 h and peaked at 12 h. Among the nine *GaHMGR* genes, the transcript levels of *GaHMGR1*, *GaHMGR6*, and *GaHMGR8* were down-regulated during the initial time points, but significantly increased at 6 h and 12 h. Additionally, *GaHMGR2*, *GaHMGR3*, *GaHMGR4*, *GaHMGR5*, *GaHMGR7*, and *GaHMGR9* expression was up-regulated soon after MeJA treatment, except that *GaHMGR5* reached the highest level at 1 h, while other genes peaked at 6 h. *GaMK1*, *GaPMK*, and *GaMVD* were all induced by MeJA and peaked at 6 h after treatment.

### Expression analysis of the MVA pathway genes in response to SA

**Figure 5:**
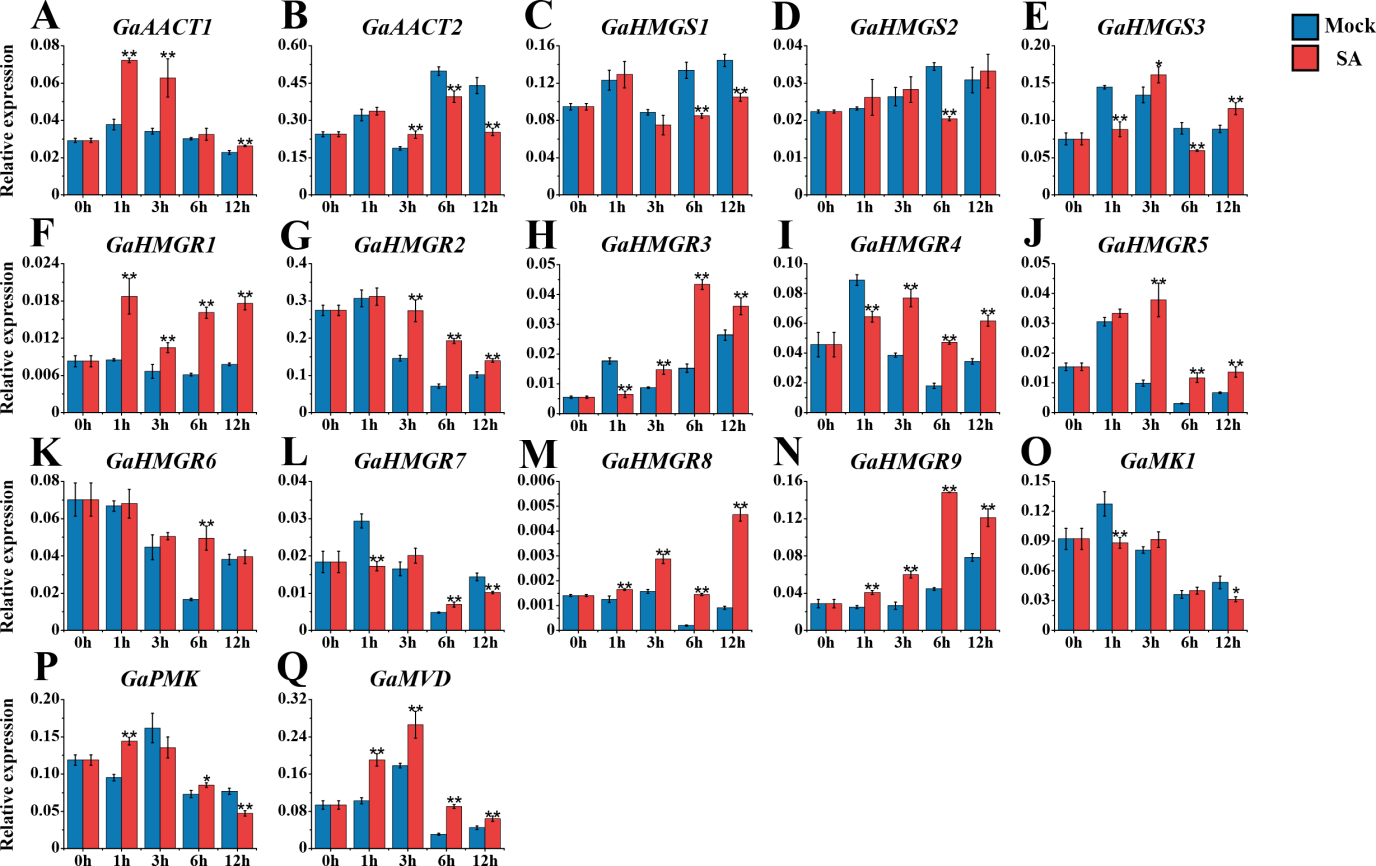
Expression analysis of the MVA pathway genes in response to SA. (A) *GaAACT1*. (B) *GaAACT2*. (C) *GaHMGS1*. (D) *GaHMGS2*. (E) *GaHMGS3*. (F) *GaHMGR1*. (G) *GaHMGR2*. (H) *GaHMGR3*. (I) *GaHMGR4*. (J) *GaHMGR5*. (K) *GaHMGR6*. (L) *GaHMGR7*. (M) *GaHMGR8*. (N) *GaHMGR9*. (O) *GaMK1*. (P) *GaPMK*. (Q) *GaMVD*. Relative expression levels were calculated by the 2^−ΔCt^ method with cotton *UBQ7* as an internal control. Error bars represent the standard deviations estimated from three independent biological replicates. Asterisks indicate significant differences between the treated and mock samples, ^∗^*P* ≤ 0.05, ^∗∗^*P* ≤ 0.01, Student’s *t*-test.

We also monitored the response of the MVA pathway genes to SA treatment (Fig. 5 and Table S8). Most genes peaked at 1 or 3 h after treatment. Compared with the mock control, *GaAACT1* expression was up-regulated and peaked at 1 h after SA treatment. *GaAACT2* expression was up-regulated at 3 h, before significantly decreasing at 6 h and 12 h. The expression levels of *GaHMGS1* and *GaHMGS2* altered slightly the first 3 h after treatment, but were down-regulated at later time points. However, *GaHMGS3* expression was up-regulated at 3 h and 12 h. Among the nine *HMGR* genes, *GaHMGR1*, *GaHMGR2*, *GaHMGR5*, *GaHMGR8*, and *GaHMGR9* were strongly induced by SA treatment, but varied in their peak timings*. GaHMGR1*, *GaHMGR2*, and *GaHMGR5* peaked early after treatment (1 h or 3 h), while *GaHMGR8* and *GaHMGR9* peaked later (6 h or 12 h). *GaHMGR3* expression was down-regulated at 1 h, followed by an increase and peaked at 6 h. *GaHMGR4* and *GaHMGR7* were down-regulated at 1 h, then increased in expression, while the expression of *GaHMGR6* only increased significantly at 6 h. The expression of *GaMK1* was significantly down-regulated at 1 h and 12 h, *GaPMK* was up-regulated at 1 h and 6 h and then decreased at 12 h, while *GaMVD* was strongly induced and peaked at 3 h.

### Expression analysis of the MVA pathway genes in response to *V. dahliae* infection

**Figure 6:**
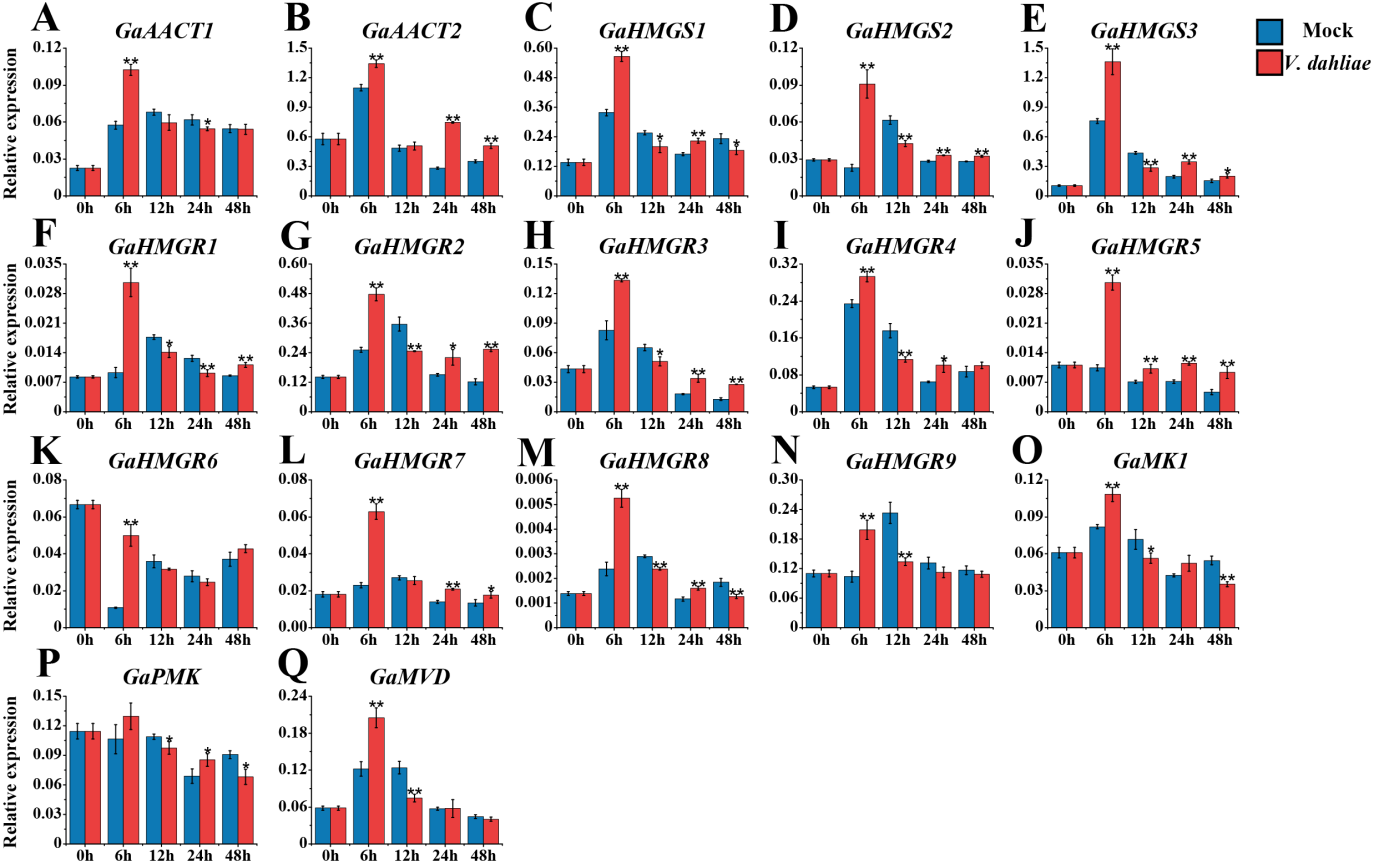
Expression analysis of the MVA pathway genes in response to *V. dahliae* infection. (A) *GaAACT1*. (B) *GaAACT2*. (C) *GaHMGS1*. (D) *GaHMGS2*.** (E) *GaHMGS3*. (F) *GaHMGR1*. (G)* GaHMGR2*. (H) *GaHMGR3*. (I) *GaHMGR4*. (J) *GaHMGR5*. (K) *GaHMGR6*. (L)* GaHMGR7*.** (M) *GaHMGR8*. (N)* GaHMGR9*. (O) *GaMK1*.** (P) *GaPMK*. (Q) *GaMVD*. Relative expression levels were calculated by the 2^−ΔCt^ method with cotton *UBQ7* as an internal control. Error bars represent the standard deviations estimated from three independent biological replicates. Asterisks indicate significant differences between the treated and mock samples, ^∗^*P* ≤ 0.05, ^∗∗^*P* ≤ 0.01, Student’s *t*-test.

The MVA pathway gene expression levels peaked quickly in *G. arboreum* after infection with *V. dahliae* (Fig. 6 and Table S8). The expression of both *GaAACT1* and *GaAACT2* was up-regulated and peaked at 6 h. However, *GaAACT1* was down-regulated at 24 h and *GaAACT2* was up-regulated at 24 h and 48 h. Three *GaHMGS* genes had similar expression patterns, with *GaHMGS1* expression decreasing at 12 h and 48 h, and *GaHMGS2* and *GaHMGS3* only decreasing at 12 h. The expression levels of all nine *GaHMGR* genes were significantly up-regulated at 6 h after *V. dahliae* infection, *GaHMGR1* expression decreased at 12 h and 24 h, then increased again at 48 h. *GaHMGR2*, *GaHMGR3*, and *GaHMGR4* were down-regulated at 12 h and then increased. *GaHMGR5* expression was up-regulated within 48 h after treatment, *GaHMGR7* was up-regulated at 24 h and 48 h, *GaHMGR8* was down-regulated at 12 h and 48 h, and *GaHMGR9* expression decreased at 12 h. *GaMK1* expression was significantly up-regulated at 6 h and then down-regulated, while *GaPMK* expression was only up-regulated at 24 h and *GaMVD* was only up-regulated at 6 h.

### Expression analysis of the MVA pathway genes in response to *H. armigera* infestation

**Figure 7:**
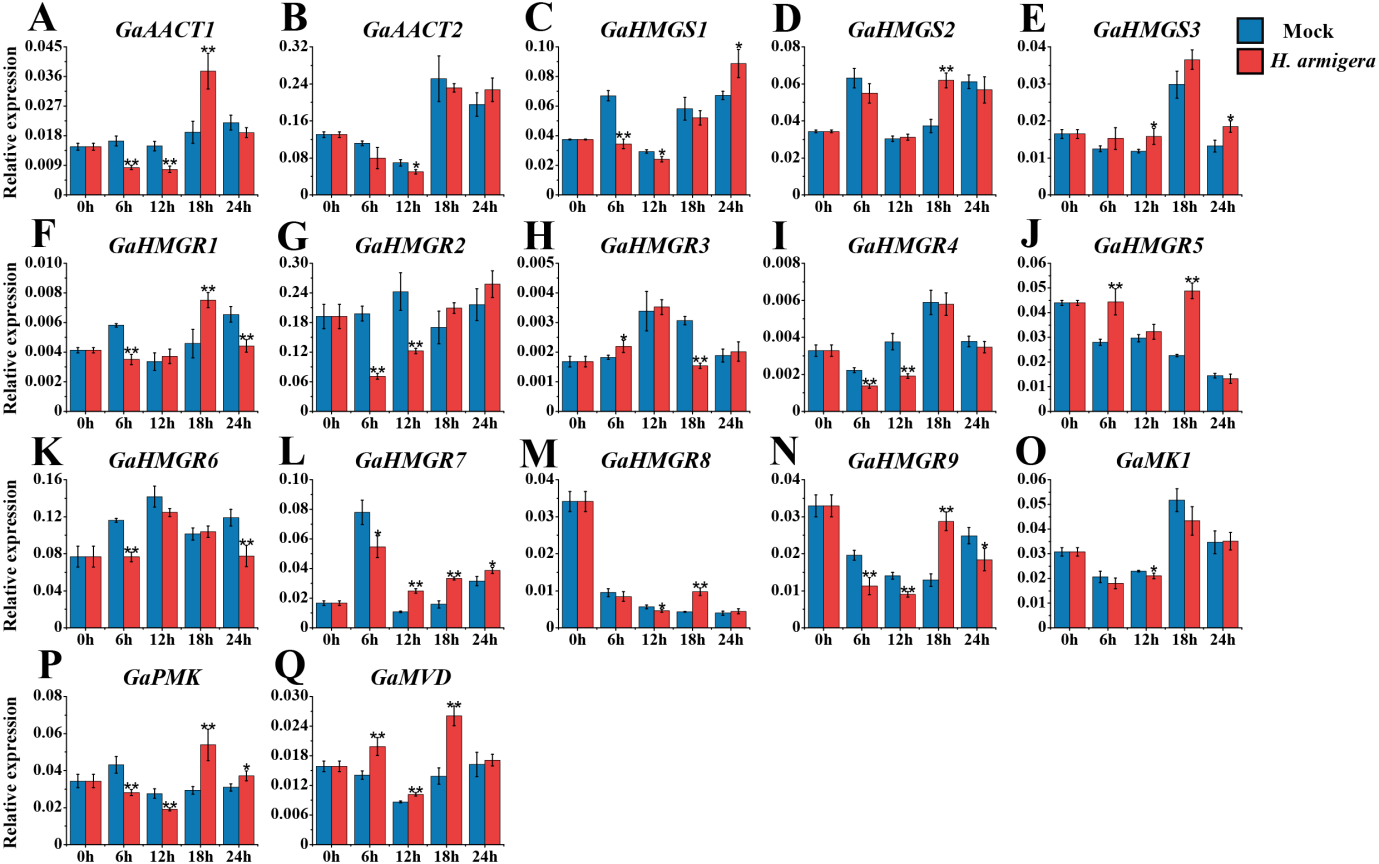
Expression analysis of the MVA pathway genes in response to *H. armigera* infestation. (A) *GaAACT1*. (B) *GaAACT2*. (C) *GaHMGS1*. (D) *GaHMGS2*. (E) *GaHMGS3*. (F) *GaHMGR1*. (G) *GaHMGR2*. (H) *GaHMGR3*. (I) *GaHMGR4*. (J) *GaHMGR5*. (K) *GaHMGR6*. (L) *GaHMGR7*. (M) *GaHMGR8*. (N) *GaHMGR9*. (O) *GaMK1*. (P) *GaPMK*. (Q) *GaMVD*. Relative expression levels were calculated by the 2^−ΔCt^ method with cotton *UBQ7* as an internal control. Error bars represent the standard deviations estimated from three biological independent replicates. Asterisks indicate significant differences between the treated and mock samples, ^∗^*P* ≤ 0.05, ^∗∗^*P* ≤ 0.01, Student’s *t*-test.

The MVA pathway genes exhibited distinct expression patterns after infestation with *H. armigera* (Fig. 7 and Table S8). Most genes responded immediately and peaked at a later time point. *GaAACT1* and *GaAACT2* expression was down-regulated soon after infestation, but *GaAACT1* expression increased significantly at 18 h. *GaHMGS1* expression was down-regulated but increased at 24 h, *GaHMGS2* was only significantly up-regulated at 18 h, while *GaHMGS3* was slightly up-regulated at 12 h and 24 h. The expression levels of *GaHMGR1*, *GaHMGR2*, *GaHMGR4*, *GaHMGR6*, *GaHMGR7*, and *GaHMGR9* were down-regulated at 6 h after infestation compared with the mock control. Subsequently, *GaHMGR1* expression was up-regulated at 18 h, following a decrease. *GaHMGR2* and *GaHMGR4* expression was down-regulated at 12 h and subsequently restored to the mock control levels. *GaHMGR6* expression decreased again 24 h after treatment, *GaHMGR7* increased after 6 h, and *GaHMGR9* was only significantly up-regulated at 18 h. The expression of *GaHMGR3* and *GaHMGR5* was up-regulated at 6 h, but *GaHMGR3* expression was down-regulated at 18 h while *GaHMGR5* was up-regulated. The expression of *GaHMGR8* was down-regulated at 12 h and up-regulated at 18 h. *GaMK1* expression showed only a slight decrease at 12 h, *GaPMK* was down-regulated in the early stages after treatment and later increased, while *GaMVD* expression was up-regulated between 6–18 h.

## Discussion

Terpenes represent the most diverse family of compounds with up to 50,000 having been identified (Vranova, Coman & Gruissem, 2013). The MVA pathway is considered to be a classical pathway for terpene biosynthesis and is ubiquitous in the three domains of life (bacteria, archaea, and eukaryotes) (Lombard & Moreira, 2011). Metabolic engineering of this pathway with the aim of enhancing terpene production has shown great potential and has been carried out in various plants (Chen et al., 2017; Liao et al., 2016; Putter et al., 2017; Ren et al., 2013).

The MVA pathway synthesizes IPP from acetyl-CoA in six enzymatic steps. In the present study, we identified two *AACT* loci, three *HMGS* loci, nine *HMGR* loci, two *MK* loci, one *PMK* locus, and one *MVD* locus in *G. arboreum*, which were unevenly distributed on eight chromosomes. Notably, an *HMGR* gene cluster containing four genes was identified on chromosome 3. SNP density analysis showed that this gene cluster was highly conserved, which is consistent with findings from our previous study (Liu et al., 2018). HMGR is the rate-limiting enzyme of the MVA pathway and is an important regulatory site for terpene biosynthesis in the cytosol (Hemmerlin, Harwood & Bach, 2012; Rodríguez-Concepción, 2006). The number of *HMGR* genes is significantly greater than that of other genes in the *G. arboreum* MVA pathway. We previously showed that *HMGR* genes underwent gene expansion in *Gossypium*, which may be related to the need to synthesize a large amount of gossypol and its derivatives in the cytosol during growth and development (Liu et al., 2018). Our current analysis of the MVA pathway gene expression levels revealed that all were expressed in our tested tissues and showed tissue-specific expression profiles except *GaMK2*. Most MVA pathway genes were highly expressed in the roots, which is consistent with the MVA pathway genes in *G. hirsutum (Tian et al., 2018)*.

Elicitors are chemicals or biofactors that can trigger physiological and morphological responses and phytoalexin accumulation. The treatment of plants with elicitors causes an array of defense reactions including the accumulation of defensive secondary metabolites (Zhao, Davis & Verpoorte, 2005). MeJA and SA are important phytohormones and signal molecules that have been implicated as abiotic elicitors of plant defenses and secondary metabolism (Robert-Seilaniantz, Grant & Jones, 2011; Vlot, Dempsey & Klessig, 2009). After treatment with MeJA and SA, the expression levels of *Ginkgo biloba AACT* and *MVK* increase, and terpene trilactones production is enhanced (Chen et al., 2017). In *Bacopa monnieri*, the related genes for triterpenoid saponin biosynthesis are induced by MeJA (Jeena et al., 2017). MeJA treatment also promotes the synthesis and accumulation of terpenes such as flavonoids in *Polygonum multiflorum* (Ho et al., 2018), celastrol in *Tripterygium wilfordii* (Liu et al., 2016), and ginsenosides in *Panax ginseng* (Choi et al., 2005). We found that the MVA pathway gene expression was responsive to MeJA, and that most genes showed a significant up-regulation, peaking in the late stages after treatment. SA is a well-known inducer of systematic acquired resistance (SAR) in plants (Gaffney et al., 1993). In *Ganoderma lucidum* (Ling-zhi), ganoderic acid (GA) biosynthesis and the transcription level of *GlHMGS* are stimulated by SA (Cao et al., 2017). In the present study, most MVA genes were induced at different time points after SA treatment, while several were down-regulated (*GaHMGS1*, *GaHMGS2*, and *GaMK1*). These results revealed the differences in expression patterns of the MVA pathway genes in response to MeJA and SA, with the response to MeJA being more intense and regular, which demonstrates the specificity of elicitors. Additionally, the gene expression induced by MeJA and SA was not completely consistent. For example, *GaHMGS1* expression was up-regulated after MeJA treatment but down-regulated after SA treatment. These variations may be due to the differences in the activation of secondary metabolic pathways in the plants by MeJA and SA.

Pathogens and herbivores alike can trigger defense responses in their host plants, and these have been attributed to a wide variety of biotic elicitors that activate specific signal transduction pathways (Paré et al., 2005). Fungal elicitors induce plants to produce secondary metabolites for defense reactions and have frequently been used to improve the production of useful plant secondary metabolites (Zhai et al., 2017). *V*. *dahliae* is a serious fungal disease affecting cotton production that induces the production of terpenes and expression of corresponding synthase genes when used to treat *G. hirsutum* cv. CCRI12 leaves (Yang et al., 2013). The transcripts of *HMGR* are induced in cultivars of both *G. hirsutum* and *G. barbadense* upon *V. dahliae* stem inoculation (Joost et al., 1995). Our study revealed that the expression of most MVA pathway genes was strongly induced 6 h after infection with *V. dahliae*, indicating that cotton plants respond rapidly to *V. dahliae* and that the MVA pathway genes play an active role in the early stages of defense. Plants respond to herbivore attack by direct and indirect defense; the former involves the release of defensive compounds to affect the feeding, growth, and survival of herbivores, while in the latter, herbivore-induced plant volatiles including terpenes are emitted to attract natural enemies of the herbivores (Aljbory & Chen, 2018; War et al., 2012). A previous study indicates that higher levels of gossypol causes a significant decrease in larval weights and moth eclosion rates of the cotton bollworm, and a delayed development of larvae and pupae (Kong, Daud & Zhu, 2010). After herbivory by lepidopteran larvae, maize releases a mixture of volatiles including terpenes that is highly attractive to its natural enemies which are females of parasitic wasps (Turlings, Tumlinson & Lewis, 1990). Maize roots release the sesquiterpene (*E*)- *β*-caryophyllene in response to feeding by the larvae of *Diabrotica virgifera virgifera*, which strongly attracts an entomopathogenic nematode (Rasmann et al., 2005). Our results showed that the expression levels of most MVA pathway genes decreased soon after infestation by *H. armigera* and were up-regulated at later stages. The MVA pathway genes elicited particular responses to *V. dahliae* or *H. armigera*, which was indicative of the role of these genes in the defense response of cotton.

## Conclusions

In conclusion, we cloned 17 potentially functional MVA pathway genes in *G. arboreum*. Expression analysis showed that the MVA pathway genes had tissue-specific expression patterns and that most genes were highly expressed in the roots. In addition, the MVA pathway genes were responsive to defense elicitors and biotic stress and most were significantly up-regulated after treatment with notable regularity.

##  Supplemental Information

10.7717/peerj.8123/supp-1 Figure S1 Chromosomal distribution of the MVA pathway genes in *G. arboreum* Chromosome numbers are displayed at the top of each bar. The scale represents megabases (Mb).

10.7717/peerj.8123/supp-2 Table S1 Primers for reverse transcription PCR

10.7717/peerj.8123/supp-3 Table S2 Primers for quantitative real-time PCR

10.7717/peerj.8123/supp-4 Table S3 Information about the MVA pathway genes from *G. arboreum*

10.7717/peerj.8123/supp-5 Table S4 The coding sequences of the MVA pathway genes in* G. arboreum*.

10.7717/peerj.8123/supp-6 Table S5 SNP density of the gene body and sequences 5 kb upstream and downstream of each MVA pathway gene in 215 *G. arboreum* accessions

10.7717/peerj.8123/supp-7 Table S6 Number of SNPs in exons and introns of each MVA pathway gene

10.7717/peerj.8123/supp-8 Table S7 Expression levels of the MVA pathway genes in various *G. arboreum* tissues tested by qRT-PCR Relative expression levels in roots, stems, cotyledons, leaves, and ovules at 0, 10, 20, 30, and 40 DPA were calculated by the 2 − Δ Ct method with cotton UBQ7 as an internal control. Results are the mean ± SD of three replicates. Statistical analysis was performed using one-way ANOVA with Tukey’s HSD test. Different letters indicate significant differences (*p* < 0.05).

10.7717/peerj.8123/supp-9 Table S8 Expression levels of the MVA pathway genes in response to MeJA, SA, *V. dahliae* infection, and *H. armigera* infestation tested by qRT-PCR Relative expression levels were calculated by the 2 − Δ Ct method with cotton UBQ7 as an internal control. Results are the mean ±SD of three replicates. Asterisks indicate significant differences between the treated and mock samples, *P ≤ 0.05, **P ≤ 0.01, Student’s *t*-test.

## Additional Information and Declarations

### DNA Deposition

The following information was supplied regarding the deposition of DNA sequences: The coding sequences are available as a Supplementary File.

### Data Availability

The following information was supplied regarding data availability: Gene primers used for reverse transcription PCR and quantitative real-time experiments are available in Tables S1 and S2. The MVA pathway members in *G. arboreum* are available in Table S3. The coding sequences of MVA pathway genes in *G. arboreum* are available in Table S4. The raw data of SNP analysis are available in Tables S5 and S6. The raw data of qRT-PCR are available in Tables S7 and S8.
