# Supplementary figures and images for: Transcriptional characterization and response to defense elicitors of mevalonate pathway genes in cotton (Gossypium arboreum L.)
Source: PeerJ. 2019 Nov 20;7:e8123. doi: 10.7717/peerj.8123 (PMC6874856; doi:10.7717/peerj.8123)

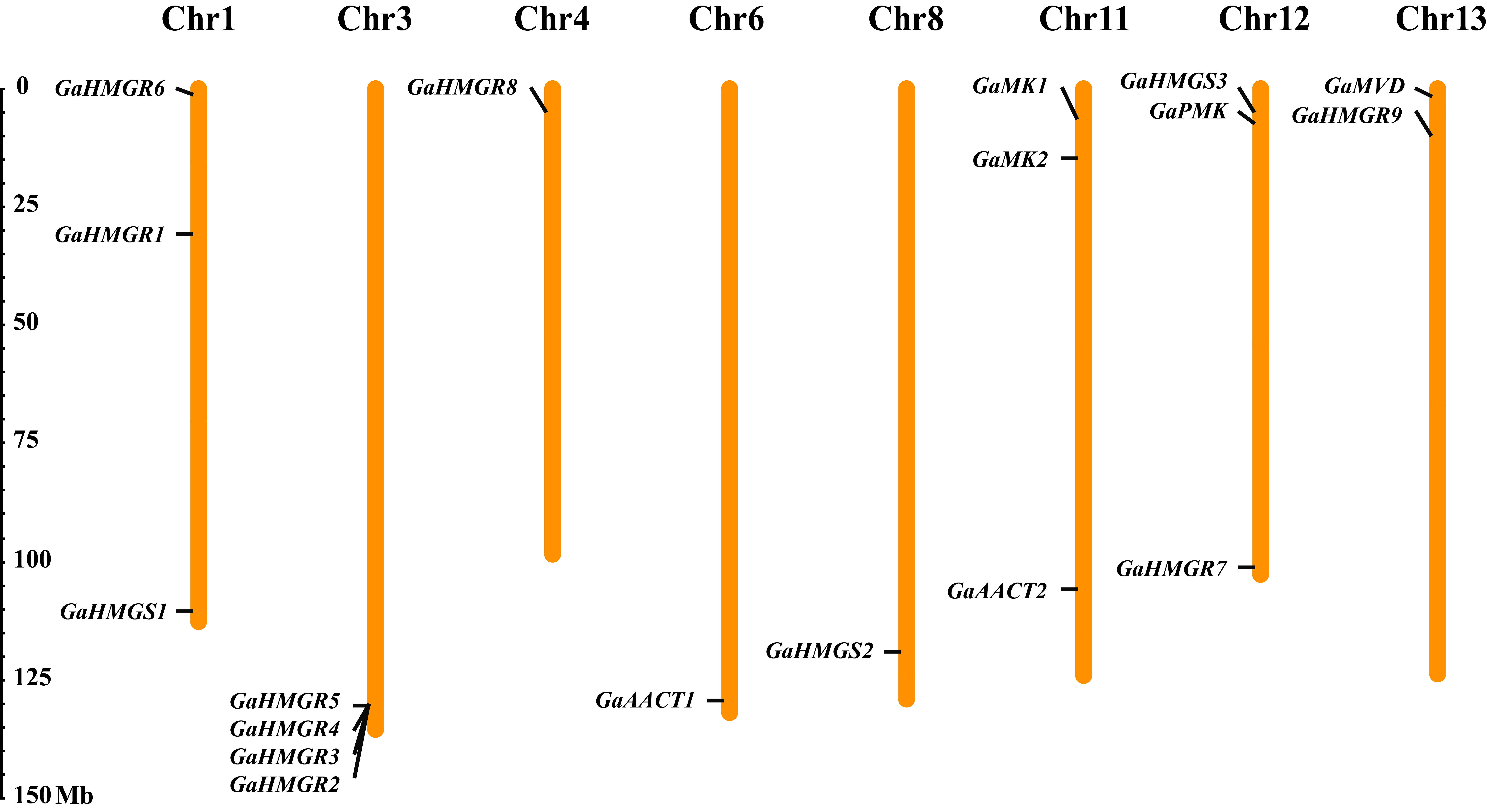

Supplement: Figure S1 — Chromosome numbers are displayed at the top of each bar. The scale represents megabases (Mb). [file peerj-07-8123-s001.png]
